# Comparative Study of Cell Nuclei Segmentation Based on Computational and Handcrafted Features Using Machine Learning Algorithms

**DOI:** 10.3390/diagnostics15101271

**Published:** 2025-05-16

**Authors:** Rashadul Islam Sumon, Md Ariful Islam Mozumdar, Salma Akter, Shah Muhammad Imtiyaj Uddin, Mohammad Hassan Ali Al-Onaizan, Reem Ibrahim Alkanhel, Mohammed Saleh Ali Muthanna

**Affiliations:** 1 Institute of Digital Anti-Aging Healthcare, Inje University, Gimhae 50834, Republic of Korea; sumon39.cst@gmail.com (R.I.S.); arifulislamro@gmail.com (M.A.I.M.); salma05.eu@gmail.com (S.A.); imtiyaj.dream@gmail.com (S.M.I.U.); 2 Department of Intelligent Systems Engineering, Faculty of Engineering and Design, Middle East University, Amman 11831, Jordan; 3 Department of Information Technology, College of Computer and Information Sciences, Princess Nourah bint Abdulrahman University, P.O. Box 84428, Riyadh 11671, Saudi Arabia; 4 Department of International Business Management, Tashkent State University of Economics, Tashkent 100066, Uzbekistan; a.muthanna@tsue.uz

**Keywords:** cell nuclei, feature extraction, prostate cancer, machine learning, segmentation, deep learning

## Abstract

**Background:** Nuclei segmentation is the first stage of automated microscopic image analysis. The cell nucleus is a crucial aspect in segmenting to gain more insight into cell characteristics and functions that enable computer-aided pathology for early disease detection, such as prostate cancer, breast cancer, brain tumors, and other diagnoses. Nucleus segmentation remains a challenging task despite significant advancements in automated methods. Traditional techniques, such as Otsu thresholding and watershed approaches, are ineffective in challenging scenarios. However, deep learning-based methods exhibit remarkable results across various biological imaging modalities, including computational pathology. **Methods:** This work explores machine learning approaches for nuclei segmentation by evaluating the quality of nuclei image segmentation. We employed several methods, including K-means clustering, Random Forest (RF), Support Vector Machine (SVM) with handcrafted features, and Logistic Regression (LR) using features derived from Convolutional Neural Networks (CNNs). Handcrafted features extract attributes like the shape, texture, and intensity of nuclei and are meticulously developed based on specialized knowledge. Conversely, CNN-based features are automatically acquired representations that identify complex patterns in nuclei images. To assess how effectively these techniques segment cell nuclei, their performance is evaluated. **Results:** Experimental results show that Logistic Regression based on CNN-derived features outperforms the other techniques, achieving an accuracy of 96.90%, a Dice coefficient of 74.24, and a Jaccard coefficient of 55.61. In contrast, the Random Forest, Support Vector Machine, and K-means algorithms yielded lower segmentation performance metrics. **Conclusions:** The conclusions suggest that leveraging CNN-based features in conjunction with Logistic Regression significantly enhances the accuracy of cell nuclei segmentation in pathological images. This approach holds promise for refining computer-aided pathology workflows, potentially leading to more reliable and earlier disease diagnoses.

## 1. Introduction

In the present time, different diseases in the human body, such as cancer, heart disease, chronic disease, brain tumors, and Alzheimer’s disease, are increasing tremendously. Segmentation of cell nuclei from the last half of the 20th century of clinical practice and academic studies has focused on the histopathology image [1]. Nuclei segmentation is a crucial task in medical image analysis and computer vision, as it is essential to numerous applications, including detecting diseases and developing new drugs. Segmentation refers to identifying and separating the objects of interest from the background. Nuclei segmentation involves separating individual cell nuclei from other structures in a tissue sample or an image. Standard traditional machine learning techniques are used for nuclei segmentation, where features are extracted from the image, and a classifier is trained to distinguish between foreground (nuclei) and background pixels. In this approach, the performance heavily relies on the quality of features extracted and the accuracy of the classifier. Despite the recent advances in deep learning techniques, traditional machine learning methods remain relevant and useful in many contexts and can provide valuable insights and results in nuclei segmentation. Machine learning (ML), a main subgroup of Artificial Intelligence (AI), refers to computational algorithms that can learn patterns from data and make predictions or decisions without being programmed [2]. ML has shown transformational capacity in various healthcare applications, including disease diagnosis, drug discovery, medical imaging, and personal remedies [3,4]. For example, ML techniques have played an important role in furthering hematological diagnostics and managing blood disorders, as in recent studies on machine learning in hematopathology. Similarly, the ML-operated model has accelerated the drug growth processes, especially in the fight against antimicrobial resistance, where traditional discovery methods face significant boundaries. These developments underline the increasing importance of machine learning in clinical decision making and improving clinical accuracy [5]. Inspired by these progressions, this study examines the use of ML algorithms to segment the cell nucleus into histopathological images, especially focusing on prostate cancer analysis to improve the reliability and efficiency of image-based diagnosis. The segmentation of nuclei in prostate tissue presents challenges different from those of other tissue types due to the specific morphological characteristics of cancer cells [6]. In malignant prostate tissue, the nuclei often exhibit notable features such as increased size, irregular shapes, and major nuclei, which vary significantly from common prostate epithelial cells. These morphological abnormalities, with the architecture of the disrupted glands and an increase in automatic congestion, make automated segmentation more complex. The exact nucleus segmentation is particularly important for diagnosing prostate cancer, as it supports Gleason grading by enabling quantitative analysis of atomic characteristics like shape, texture, and density [7]. By dividing the nucleus properly, pathologists can assess malignant levels more frequently and fairly, assisting in the initial identification and treatment plan. Therefore, as proposed in this study, integrating strong machine learning-based division methods is important in increasing the accuracy and efficiency of computer-aided diagnostic systems for prostate cancer.

The main goal of every segmentation technique is to separate the foreground and background of an image. These techniques can be divided into two categories: (1) based on optimization, where a cost function for energy is maximized or minimized, e.g., active contours, level set, global minimizers, graph-based; (2) based on machine learning, where a computer or network is trained to distinguish features, such as deep convolutional neural networks, which have drawn much attention. Accurate cell identification can facilitate research into how cells respond to different treatments. Nuclei segmentation can ensure that patients receive better care, and it can hasten medication discovery and treatment procedures.

Segmenting nuclei for a particular tissue type is challenging. Therefore, computational pathology [8] and microscope images are important tools for diagnosing diseases, since these photographs may convey much information for computer-aided diagnosis (CAD). Cell types and staining variations influence the visual characteristics of stains. Recent research has shown that deep learning algorithms are very effective for segmenting biological data in a complex way.

We used machine learning for cell nucleus segmentation. Traditional methods such as Otsu’s thresholding and watershed are often ineffective due to noise, stain variation, and their sensitivity to overlapping nuclei in histopathology images. They rely on certain rules that cannot be compatible with complex tissue structures. Conversely, the machine can learn from learning data, handle diverse morphological variations, and can remove meaningful patterns using computational features. This research addresses the difference by comparing machine learning models to achieve more accurate and strong nuclei segmentation, especially in prostate cancer analysis, with handcrafted and CNN-based characteristics. We describe how different machine learning methods were used to assess the quality of pathological image segmentation and compare different ML approaches. Manually recognizing and annotating medical images is a timely and labor-intensive task. Research into computer-aided medical image segmentation has flourished in recent years. This is a great benefit of the extending collaboration between artificial intelligence and medical image analysis. Computer-aided segmentation allows clinicians to create image markers relevant to the illness treatment process quickly and easily, which enables them to discover malignant tissue affected by prostate cancer (PCa) in an early stage. Pathologists may assist more patients with this strategy while still providing accurate diagnoses. In addition, nuclei segmentation can yield information about the shape of the gland, which is important for grading cancer [9]. We show the proposed pipeline where the workflow of nuclei segmentation was divided into seven phases. Our procedure is carried out at the image patch level. An entire slide tissue picture is separated into patches. Four categorization methods are used and compared: Logistic Regression, Support Vector Machine (SVM), K-means, and Random Forest (RF) for performance improvement and efficiency. We conducted experiments using two prostate cancer datasets, one publicly available from Radboud University Medical Center and another publicly available MoNuSeg dataset.

**Figure 1:**
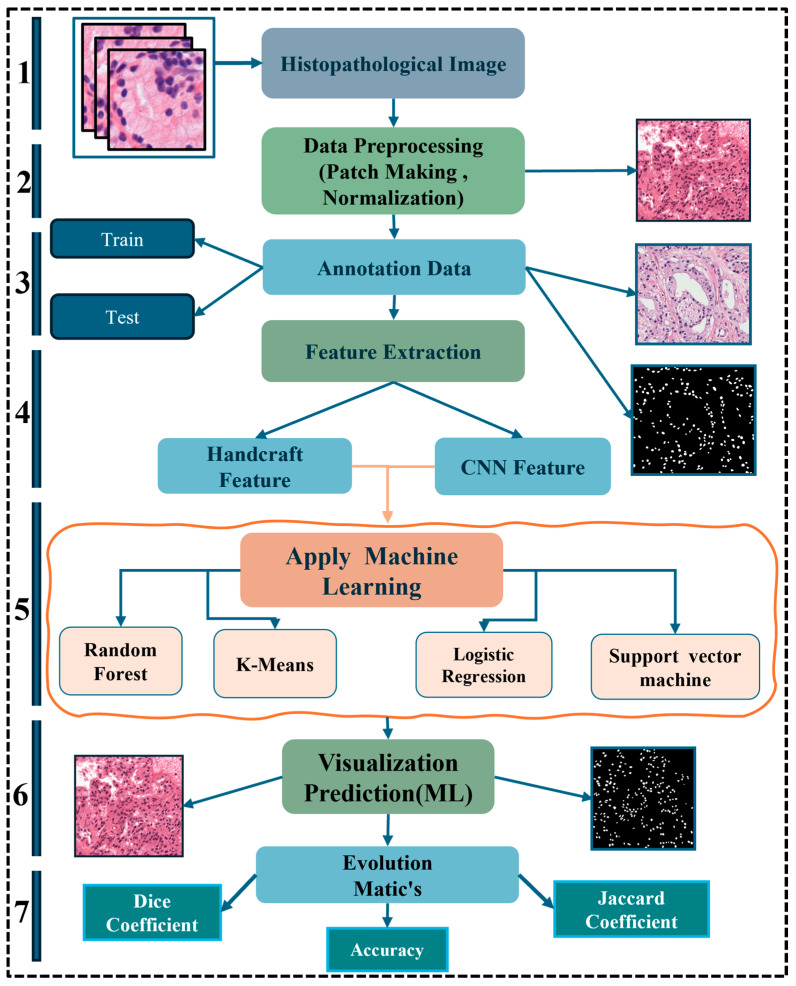
The proposed pipeline for prostate cancer nuclei segmentation in histopathology sections.

Logistic Regression has the best segmentation results in our experimental study. Still, it takes a long time to process, with the Jaccard Index of 55.61 and Dice coefficient of 74.24 with 96.90 accuracy for the CNN feature, and the Jaccard Index of 53.81 and Dice coefficient of 68.32.24 with 96.10 accuracy for the handcrafted feature. The methodology section describes feature extraction mechanisms using a pre-trained VGG-16 model using ImageNet weights with Logistic Registration, Random Forest, K-means, and Support Vector Machine (SVM) for histopathological image segmentation. Figure 1 illustrates the proposed pipeline of our study, comprising the following seven sequential steps: (1) Histopathology image data acquisition, (2) Data preprocessing, (3) Manual nuclei annotation, (4) Feature extraction, (5) Application of machine learning algorithms, (6) Model prediction, and (7) Evaluation metrics and comparison.

## 2. Literature Review

Nuclei segmentation is an important task in medical image analysis, and traditional machine-learning methods have been widely applied to this problem. The accuracy of nuclei segmentation can be improved by various techniques, including thresholding, region growing, active contours, random forests, and clustering algorithms. Islam Sumon et al. [10] applied a computer-based nuclei segmentation and detection approach through adaptive thresholding to detect cells and achieved promising accuracy. Within the various machine learning algorithms, SVMs are the prominent performers for classification on many segmentations and histopathological image classification, blood cell detection, tissue segmentation [11], segmentation of brain tumor portions [12], and nuclei segmentation from histopathology [13]. These methods have been applied to various medical imaging modalities, including confocal microscopy, histopathology, and cytology images. Many traditional machine learning approaches have achieved state-of-the-art performance in nuclei segmentation. Ikromjanov et al. [14] used Support Vector Machines based on Laplace edge features to analyze the histopathological tissue image, and they achieved the promise of accuracy for classification and nuclei cell detection. Haq et al. [15] introduced a watershed algorithm and morphological operations for separating tissue cells and then finding the cell classification using a neural network classifier by feeding the cells.

Some unique related works include multiscale CNNs, adaptive thresholding, and a combination of random forests and active contours for nuclei segmentation. These methods have contributed to advancing the field of nuclei segmentation and provided insights into the development of accurate and efficient segmentation algorithms. Arsa Wan Han et al. used the VGG-16 feature with a Random Forest classifier for batik classification, which is superior to the current technique’s use of color and texture features [16]. Sumon et al. used deep learning to identify the best segmentation scale(s) for predicting land cover classes based on potential image segmentation alternatives [17]. Karimi et al. proposed a patch-based approach that detects the significant patch from the whole slide image and then applies CNN feature extraction to classify the patch image, which is the CNN-based feature extraction method [18]. In the last few years, many surveys have been conducted on different methods of computer vision technologies in medical image processing [19]. Researchers have described preprocessing methods, nuclei classification, segmentation, detection, and preprocessing techniques [20]. Several traditional methods have been used for segmenting nuclei in histopathological images. The methods range from simple background subtraction to more complex ones, such as marked point processes [21]. Identifying cells in 3D, label-free, or thick tissue sections is challenging, especially if they overlap, touch, or have non-conventional morphologies, intensities, or patterns.

There has been progress in overcoming these problems through international competitions, but there is still a need for a more general solution [22,23]. Nuclei morphology analysis, in particular, has a crucial role in identifying breast cancer. Early research used gradient and intensity approaches to choose feature points in the immediate area using gradient magnitude maps and Euclidean distance [24,25,26,27] for nuclei detection and segmentation. Still, this approach is used to detect the nuclei accurately. Gradients, global textures, and shape information are combined with handcrafted features and computational features to train machine learning methods such as SVM, Random Forest, and Logistic Regression [28,29,30,31]. Jagannathan et al. [32] introduced an improved F-score method to improve the ability to select features, and this method achieves higher accuracy for breast cancer classification. Wang et al. [33] applied Support Vector Machines (SVMs), K-Nearest Neighbors (KNNs), Multilayer Perceptrons (MLPs), Gaussian Naive Bayes (GB), and Classification Trees, and compared five nonlinear machine learning techniques. Kollem et al. showed a comprehensive description of segmentation techniques with thorough explanations of each method [34]. Due to high variability and low contrast in medical images, it is not easy to segment images efficiently. Gayathri et al. proposed deep learning and machine learning (ML) algorithms, features that can be extracted from fundus images and categorized according to severity [35]. Global and local feature extraction from images is performed using a Multipath Convolutional Neural Network, according to MI Razzak et al. [36]. For contour-aware segmentation, we used a fully conventional network, whereas for classification, we used an extreme machine learning approach based on CNN features [37]. A least squares Support Vector Machine-based approach to segmenting color images is presented. In the Gabor filter, the texture features of an image pixel are represented as Maximum local energy, Maximum gradient, and Maximum second-moment matrix [38]. Shin et al. analyzed convolutional neural network (CNN)-based classification models. To classify the images, the acquired features were fed into machine learning algorithms such as the K-Nearest Neighbor algorithm (K-NN), the Support Vector Machine (SVM), and the Random Forest algorithm (RF). Hoo-Chang Shin combined hybrid clustering and Logistic Regression to find a nonlinear decision boundary to classify tumors and edema [39].

Ruusuvuori et al. proposed a regularized Logistic Regression classifier that generates many artificial features [40]. This study’s performance for two use scenarios demonstrates that segmentation outcomes are reliable even for straightforward models with significant sparsity. We implemented a completely automated magnetic resonance (MR) image’s right ventricle segmentation technique. An MR test image is first over-segmented into super pixels, and then each superpixel is examined using Random Forest (RF) classifiers to find the existence of affected areas [41,42].

## 3. Materials and Methods

### 3.1. Image Acquisitions

**Figure 2:**
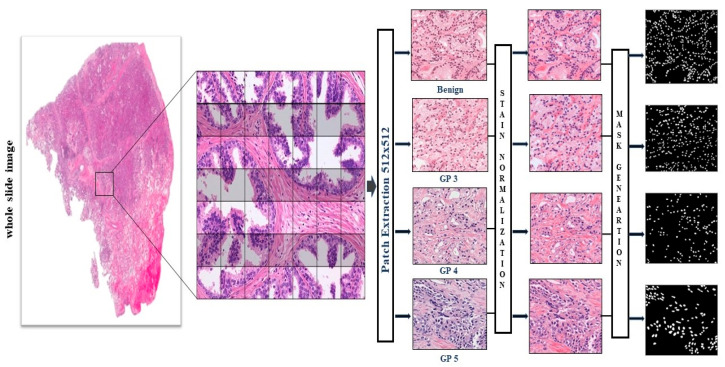
Dataset preparation: there are four types of prostate cancer histopathology images from the Kaggle and the Severance Hospital. There are two types of images: benign and malignant. Tumors of Grade 2 are considered benign, and Grades 3, 4, and 5 are considered malignant. In this paper, we focus on Grade 3, Grade 4, and Grade 5. In terms of the MoNuSeg dataset, we consider breast cancer, stomach cancer, and liver cancer.

We utilized two distinct datasets obtained from two distinct hospitals. We used publicly available data from a public dataset. In the Netherlands, Radboud University Medical Center prepared the public dataset. The 3Dhistech Panoramic Flash II 250 scanners were used to scan the slides at 20× magnification. We chose 50 whole slide images of prostate cancer tissue from the Radboud dataset and produced 2900 image patches of size 512 × 512 pixels. Another publicly accessible dataset called Multi-organ Nucleus Segmentation (MoNuSeg) was acquired. It may be accessed at https://monuseg.grand-challenge.org/Data. Additionally, from the MoNuSeg dataset, we used various cancer types such as breast, abdomen, and liver, and used 20 whole slide images from which we made 1400 image patches of the same size. This dataset was produced by downloading a tissue image stained with H&E and magnified 40 times from the TCGA database. A standard procedure to improve a tissue section’s contrast is H&E staining, which is frequently used for tumor assessment (grading, staging, etc.). We made a patch image size 512 × 512 pixels from the whole slide image, shown in Figure 2.

### 3.2. Patch Generation from Whole Slide Image

A novel patch generation method was developed to accurately segment the cell nuclei from a microscopic biopsy image. After the images were preprocessed (i.e., applying filtering, gamma correction, and sharpening methods), patches with target size 128 × 128 were extracted from a single image. In detail, the image is viewed from the top left corner ($p_{x}$, $p_{y}$), and the shifting of the sliding window ($i_{x}$, $j_{y}$) across the top and down the left was carried out with grid spacing $i_{x}$ = $j_{y}$ = 64 along row and column. After training the model, the patches generated from a single image were fed to the network for testing. Further, the predicted segmented patches of size 128 × 128 were merged to create the original size of the image and visualize the segmented result.

### 3.3. Color Normalization

Histopathology images stained with H&E exhibit significant color variations since stains are manufactured differently, different staining techniques are employed, and various digital scanner response mechanisms are used. When CNN-based computational pathology methods are applied across domains, stain differences can be a major problem. The performance of CNN-based methods can be greatly improved when the images are normalized [43].

**Figure 3:**
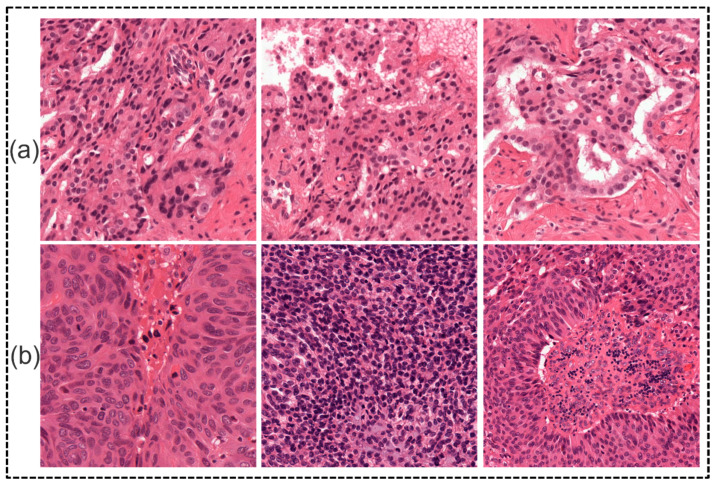
Results of stain normalization. (**a**) Samples of Radboud University. (**b**) Samples of Yonsei University.

However, because the two stains can be normalized against one another, normalization techniques created for conventional vision applications only offer limited advantages in computational pathology applications [44]. To translate networks trained on data from one site to another, from other places, or to normalize pathological images, several approaches have been reported [45,46,47,48]. We empirically found that sparse stain normalized H&E images following Ref. [49] performed better with our nuclei segmentation method. We empirically discovered that our nuclei segmentation algorithm performed better with sparse stain-normalized H&E images [50]. Examples of pictures with normalized stains are shown in Figure 3.

### 3.4. Feature Extraction-Based Segmentation

This study extracted the feature using handicrafts and CNN-based methods to increase the nucleus division. The features made by using filters such as Sobel, Gabor, and LBP occupy the edge, texture, and intensity characteristics. In contrast, CNN-based features were extracted using a pre-trained VGG-16 model to receive deep hierarchical representations. These features were input for various machine learning algorithms to evaluate partition performance.

#### 3.4.1. Handcrafted Feature

Histopathological images undergo preprocessing procedures like normalization, scaling, and noise reduction before feature extraction. The histopathological image $I\in R^{H\times W\times C}$ acts as the input for manually created feature extraction. In this study, 42 handcrafted features were extracted from histopathological images to support nuclei segmentation. These characteristics are spread over several categories, including edge detection, texture, intensity, frequency, and size details. For edge-based analysis, five filters were applied to catch the magnitude and shape of the nucleus structure, such as Canny, Sobel, Roberts, Scharr, and Prewitt filters. Two noisy techniques, Gaussian and median filtering, were used to smooth images, preserving important details. A Laplacian filter was employed to increase structural boundaries. Six Gabor filter features were calculated using various tilts and wavelengths to catch the texture and frequency characteristics. Additionally, six intensity-based histograms were calculated to represent the pixel intensity distribution within Pisces, mean, standard deviation, obliqueness, kurtosis, and entropy-nuclei areas. The local binary pattern (LBP) method contributed eight features by encoding the local texture pattern around each pixel. Finally, six features of morphological size were extracted, the area, circumference, eccentricity, length of the major axis, minor axis, and solidity, providing significant insight into nuclear geometry. Together, these handcrafted features provided a diverse and informative representation of nucleus structures, supporting the division performance of the machine learning algorithms. A representation of the extracted features is set as:

(1) $$F_{HC}=\{F_{1},F_{2},\ldots \ldots ,F_{42}\}$$

where each $f_{i}$ represents a specific feature derived from one of the applied filters. We used several edge detection filters. The Canny Edge filter detects strong and weak edges. Gradients are calculated along the x and y directions using the Sobel filter. Diagonal edges are detected via the Roberts filter. We increased the accuracy of edge detection with Scharr and Prewitt filters.

(2) $$f_{edge=}\sum_{x,y}\left|\nabla l^{\prime }\left(x,y\right)\right|$$

where $\nabla l^{\prime }$ represents the gradient magnitude computed using edge detection operators. The Gaussian and median filters eliminate noise while maintaining key structural elements.

(3) $$l_{G}=l^{\prime }*G\sigma ,\;l_{M}=\mathrm{median}(I^{\prime })$$

where *Gσ* is a Gaussian kernel with standard deviation *σ*, and $l_{M}$ is the median-filtered image.

The Laplacian filter draws attention to glandular structures and improves contrast.

(4) $$L\left(l^{\prime }\right)=\nabla^{2}l^{\prime }$$

where $\nabla^{2}l^{\prime }$ is the second derivative of the image.

Textural properties, including cell size, shape, and orientation, can be extracted using Gabor filters.

(5) $$G_{\lambda ,\theta ,\Psi ,\sigma ,\gamma }(x,y)=\exp\left(-\frac{x^{\prime 2}+\gamma^{2}y^{\prime 2}}{{2\sigma }^{2}}\right)\cos\left(2\pi \frac{x^{\prime }}{\lambda }+\Psi \right)$$

where $x^{\prime }$ and $y^{\prime }$ are rotated coordinates and parameters λ, θ, Ψ, σ, and γ control frequency, orientation, phase, standard deviation, and aspect ratio, respectively. We applied several textural feature extraction methods for textural features such as histogram-based features and Gray Level Co-occurrence Matrix (GLCM) for contrast, correlation, energy, and homogeneity features. We extracted the Local Binary Pattern (LBP) for pattern recognition by the following equation.

(6) $$LBP(x,y)=\sum_{p=0}^{P-1}s(l_{p}-l_{c})2^{p}$$

where $l_{c}$ is the central pixel intensity, $l_{p}$ are neighborhood pixel intensities, and s(x) is a step function. After extracting the features, we then combine all of the extracted handcrafted features into a feature vector using the following equation $F_{HC}^{\prime }={S(F}_{HC})$.

#### 3.4.2. CNN Feature

Feature extraction using deep neural networks is another popular approach to analyzing histopathological images. The VGG-16 pre-trained demonstration of profound neural network design is broadly utilized for image classification and segmentation. In this experiment, the VGG-16 pre-trained model extracts features from histopathological images by passing them through the model’s layers. We employ three VGG-16 pre-trained models as a feature extractor. VGG-16 was initially trained using the 1,000,000 image ImageNet dataset. There are 16 hidden levels in this network, and each layer contains fully connected layers, convolutional layers, and layers with maximum pooling.

The histopathological images that make up the input data are elements of double-struck cap R to all photos that make up the input data $I\in R^{H\times W\times C}$, where H and W denote the height and width of the image, respectively, and C represents the number of color channels. Additionally, binary segmentation masks the element of the superscript base, open brace 0,1 close brace, end base, to the segmentation masks $M\in {\left\{0,1\right\}}^{H\times W}$ are used for training and evaluation. Each image undergoes preprocessing, including normalization and resizing to $I\in R^{128\times 128\times 128}$, ensuring compatibility with the VGG-16 model.

Three different VGG-16 models that have all been pre-trained on ImageNet are used. Each model’s many convolutional layers yield feature maps.

Model 1 extracts features from the layer $l_{1}$, yielding a feature map $F_{1}\in R^{H_{1}\times W_{1}\times D_{1}}$.

Model 2 extracts features from the layer $l_{2}$, yielding a feature map $F_{2}\in R^{H_{2}\times W_{2}\times D_{2}}$.

Model 3 extracts features from the layer $l_{3}$, yielding a feature map $F_{3}\in R^{H_{3}\times W_{3}\times D_{3}}$.

Extracted features are represented as the following equation.

(7) $$F_{i}=VGG16_{l_{i}}\left(l^{\prime }\right),\;i\in \{1,2,3\}$$

where $F_{i}$ is the feature tensor obtained from the $l_{i}$ layer of the corresponding VGG-16 model. We use a feature selection method, S, like Principal Component Analysis or mutual information-based selection, to eliminate redundancy and choose the most useful features: $F_{s}=$ *S* ($F_{c}$), where $F_{s}\in R^{H_{1}\times W_{1}\times D_{1}}$ is the reduced feature set used for segmentation.

**Figure 4:**
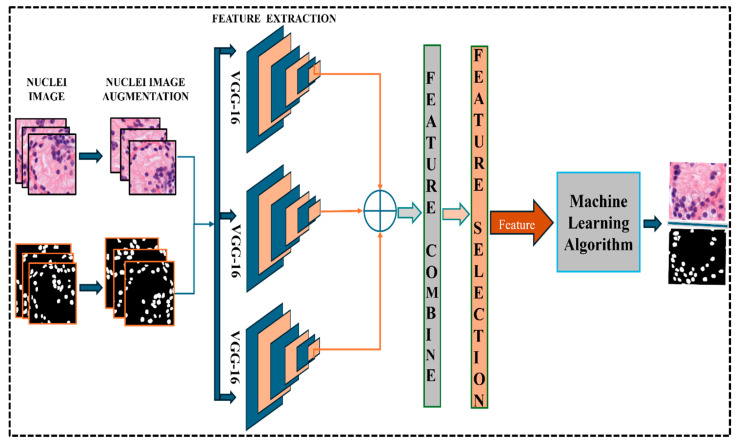
Pre-trained VGG-16 model for feature extraction.

Using traditional machine learning algorithms directly on raw pixel data can be challenging because images often contain many pixels. Input data can be reduced in dimension by using CNN features as input to an ML classifier while retaining important image information. Using CNNs for feature extraction and ML algorithms for segmentation allows us to achieve greater flexibility in finding the most suitable classifier for the given segmentation task. We can try different CNN architectures and hyperparameters, and we can also try different machine learning algorithms. Figure 4 shows the Pre-trained VGG-16 model for feature extraction.

### 3.5. Machine Learning Segmentation Algorithm

In this paper, we applied four machine learning algorithms: Random Forest, SVM, Logistic Regression, and the K-means algorithm.

#### 3.5.1. Random Forest

**Figure 5:**
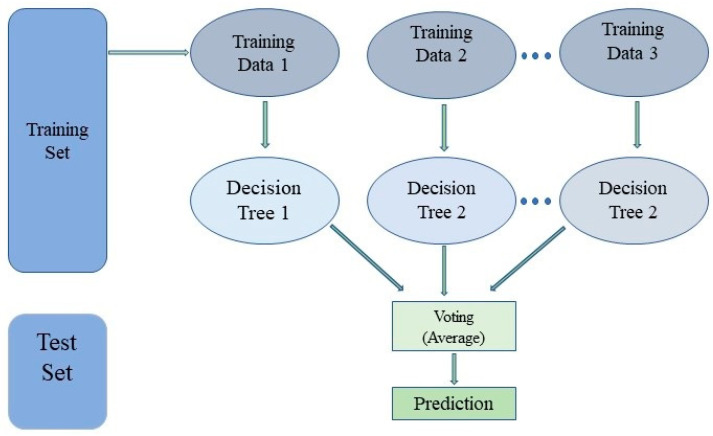
Architecture of the Random Forest algorithm.

Random Forest belongs to the supervised learning methodology of the machine learning approach. As the name implies, Random Forest is a classifier that creates numerous decision trees based on the dataset’s features to increase prediction accuracy. The Random Forest employs predictions from each tree instead of relying on just one, and it predicts the outcome based on the votes of most forecasts. As a result, each tree that makes up Random Forest has a different subset, the terminal nodes are then chosen for the last process, and the categorization outcome is determined by a majority vote, as shown in Figure 5. The fundamental rule of Random Forest is the minimal Gini index defined in Equation (8).

(8) $$\mathrm{Gini}(m)=\sum_{k=1}^{N}x_{n_{i}}$$

(9) $$x_{n_{i}}=\frac{L_{n_{i}}}{L}$$

where *N* and $x_{n_{i}}$ have a relationship between class $n_{i}$ and the number of classes and the likelihood function of the data node m. L has precisely the same number of trees as Random Forest, and $L_{n_{i}}$ denotes the class n’s tree count.

**Figure 6:**
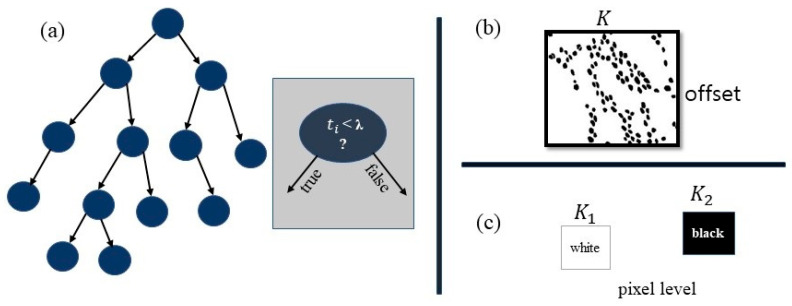
Decision tree and node function (**a**) decision tree structure, (**b**) feature representation, (**c**) pixel-level class labels.

In the Random Forest algorithm for nuclei segmentation, we consider a binary tree (Figure 5) with the structure of a decision tree. With the labeled training data, starting at the root, it is discovered that the function t and λ threshold optimize the information gain. Then, down nodes are considered as children (Figure 6), with a linear classifier at the output level and background. For a pixel in position t, the node function $t_{i}$ is demonstrated in Equation (3).

(10) $$t_{i}=\sum_{mℇ℘}^{i}x_{m}.y_{m}$$

where k indexes one or two rectangles as described in Figure 6b,c (i.e., ℘={0} or {0,1}). Here, $x_{k}$ and $y_{k}$ represent all numbers of white and black pixels, respectively.

The Random Forest model for nuclei division was trained using 100 decision trees. The maximum depth of each tree to prevent overfitting was 20. Sovereign impurities were used as partition criteria to reduce class impurities. For each node, the number of facilities assumed was the square root of the total facilities. Bootstrap sampling was applied to increase model diversity. A minimum of 2 samples were required to divide a node, and 1 per leaf node. A random status of 42 was used to ensure breeding. Random Forest, being non-parametric, does not use specific loss functions. Reducing the guinea impurities during training improved its performance.

#### 3.5.2. Support Vector Machine (SVM)

Classification and regression issues can be addressed using the “Support Vector Machine” (SVM) supervised machine learning method. N features are in N-dimensional space (feature number indicated by N). The Support Vector Machine approach looks for a hyperplane that classifies the data points.

**Figure 7:**
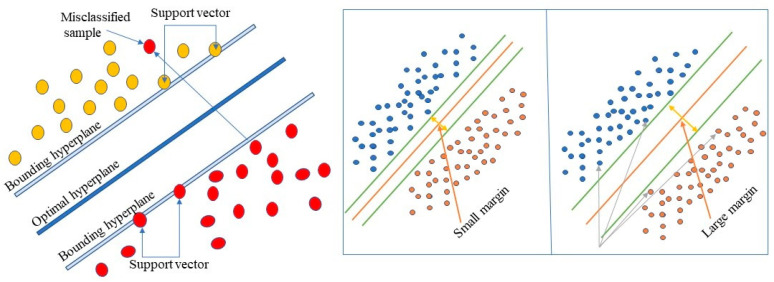
Support vector machine (SVM) and data point separation margin.

In Figure 7, boundary hyperplanes are used to split the two classes of data points, background and foreground, with a margin to separate data points for both classes, optimal hyperplane maintenance, and distance between both data classes. Increasing the marginal distance allows the SVM to increase the categorization confidence for subsequent data points. Decision boundaries, known as hyperplanes, are used to organize data points, as demonstrated in Figure 7. Different classifications can be given to data points on either side of the hyperplane.

In this experiment, we used Support Vector Machines (SVM) for nuclei segmentation from histopathology images. The SVM model is trained using a set of labeled training data extracted by the VGG-16 pre-trained models, where each pixel or R.O.I. is labeled as either nucleus or background. The SVM model learned to classify new, unseen pixels or ROI as either nucleus or background based on their extracted features. This experiment used a Support Vector Machine (SVM) with a radial basis function (RBF) kernel for nucleus segmentation from histopathology images. SVM is a supervised machine learning algorithm that forms an optimal hyperplane to isolate data points in n-dimensional space. The RBF kernel was chosen due to its effectiveness in handling non-linear separation, which is common in biological image data. The model was trained on VGG-16 pre-inf. We used a regularization parameter C = 1.0 and gamma = ‘scale’ to control the flexibility of kernels for training. These parameters were selected based on performance during verification to avoid overfitting and improve generalization. The SVM model then learned to classify the unseen ROI based on extracted deep features.

#### 3.5.3. Logistic Regression

The supervised machine learning technique, Logistic Regression, completes binary classification problems using probabilistic predictions. The model produces a binary or dichotomous result with only two potential results. In this paper, the nuclei segmentation prediction result is a binary foreground and background. Logical Regression, which investigates the connection between one or more independent variables, divides data into distinct groups. The model determines a given incident’s mathematical likelihood of falling into a certain category.

This paper’s output represents a binary class of zero or one. The detection nuclei pixel represents one (1) as white, and the background pixel represents zero (0) as black. Logistic Regression employs a logistic function, known as a sigmoid function, to map predictions and their probabilities. Additionally, we suppose the result of the sigmoid function indicates that the instance belongs to that class. In that case, the model predicts that the estimated probability is greater than a predetermined threshold on the graph. An example is expected not to belong to a class if its estimated probability falls below the predetermined threshold in Logistic Regression using the sigmoid function as the activation function, shown in Equation (11).

(11) $$f(x)=\frac{1}{1+e^{-1}}$$

where e = base of natural logarithms, and value = the number value you want to change. The Logistic Regression is represented by Equation (12).

(12) $$Y=\frac{e^{{(a}_{0}+a_{1}x)}}{1+e^{{(a}_{0}+a_{1}x)}}$$

where x = input value, y = output level, $a_{0}$ = bias, and $a_{1}$ = coefficient value for linear regression. We input values linearly to predict output values using weights or coefficient values. Unlike linear regression, this output value has a binary value (0 or 1) rather than a numerical value. The model uses a sigmoid activation function, which replaces the input features from 0 to 1 with a potential output. A classification range of 0.5 was applied, where more than 0.5 outputs were classified as a nucleus and the background. The training process was performed with gradient lineage adaptation with a learning rate of 0.01, and regularization techniques were used to prevent overfitting. The training was conducted at the age of 100, ensuring convergence with a maximum of 1000 ages. The binary cross-entropy log function was used as a loss metric, effectively measuring the difference between the estimated possibilities and the real binary label. Model parameters, including weight and prejudice, were re-updated during each era to reduce losses.

#### 3.5.4. K-Means Clustering

**Figure 8:**
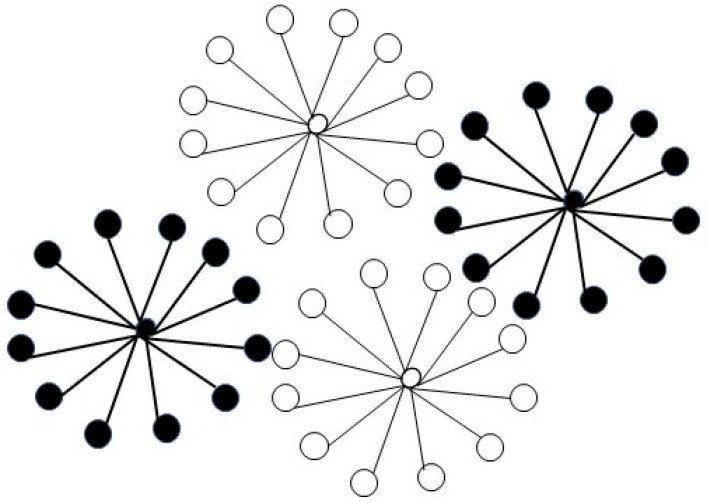
K-means clustering algorithm.

In nuclei segmentation, the unsupervised K-means clustering technique is used to separate the area of interest from the background. The given data are clustered or divided into K-clusters or sections based on the K-centroids. Groupings were found based on similarities between data and the group members represented by K, as shown in Figure 8.

This experiment performs a thresholding operation to convert the grayscale histopathology image into a binary image where the pixels are classified as foreground (nuclei) or background. This is typically achieved by selecting a threshold value that separates the nuclei from the background. Each pixel or area of interest in a histopathology image has a collection of characteristics extracted from it.

Common features for nuclei segmentation include shape, size, texture, and intensity. The K-means clustering method receives its input from the retrieved features, which group the pixels or ROIs into K-clusters based on their similarity in feature space. The centroids of each cluster are calculated and represent the characteristic features of that cluster.

## 4. Results

The network was created using the TensorFlow DL framework. The Keras and TensorFlow libraries were used in the Python 3 programming language to build the DL and ML models. The models were designed, validated, and tested on a PC using the following variables: an Intel core 5 CPU (2.5 GHz), one NVIDIA GeForce R.T.X. 3070ti GPU, and 32 GB of RAM.

This article focuses on prostate cancer nuclei segmentation based on AI. However, the best segmentation results were achieved using the CNN-based features and Logistic Regression algorithms, with values of 74.24 and 55.61 for the Dice coefficient and Jaccard coefficient, respectively, with an accuracy of 96.90 in Table 1. The handcrafted Logistic Regression algorithm achieved the best performance of 68.32 and 53.81 Dice coefficient and Jaccard coefficient, respectively, with an accuracy of 96.10 in Table 2.

**Figure 9:**
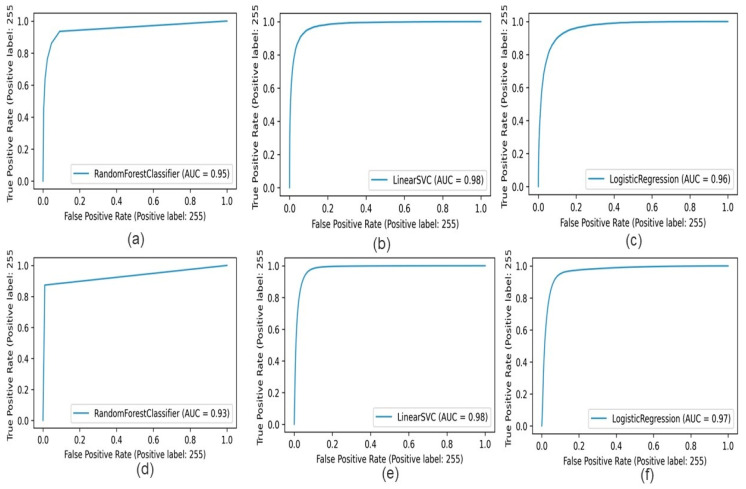
Training accuracy of (**a**) Random Forest, (**b**) Support Vector Machine, (**c**) Logistic Regression with CNN feature, (**d**) Random Forest, (**e**) Support Vector Machine, and (**f**) Logistic Regression with handcrafted features.

**Figure 10:**
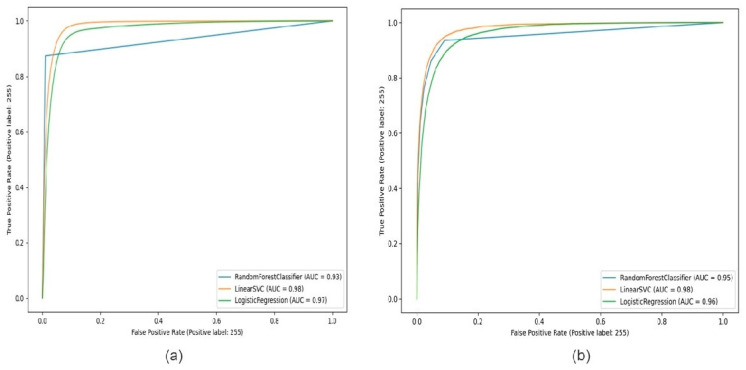
Comparison of accuracy (**a**) Random Forest (blue), Support Vector Machine (orange), Logistic Regression (green) with CNN features, and (**b**) Comparison of Random Forest (blue), Support Vector Machine (orange), and Logistic Regression (green) with handcrafted features.

The learning graph, shown in Figure 9, demonstrates Random Forest, SVM, and Logistic Regression registration accuracy curves, with CNN and handcrafted features in the learning graph of Figure 9a. The comparison results of Random Forest, SVM, and Logistic Regression registration for the CNN feature appear. Figure 9b shows the comparison result of Random Forest, SVM, and Logistic Regression for the handcrafted features. Figure 10 shows that apparent Support Vector Machine accuracy is the height for the bot feature method, but Logistic Regression has the best performance for segmentation results in both feature methods.

Segmentation accuracy is a metric that measures the performance of a segmentation model for training and testing datasets. It is derived by dividing the number of pixels in the ground truth (reference) segmentation by the number of precisely segmented pixels. The result is expressed as a percentage, with 100% indicating perfect segmentation accuracy. It is frequently used in computer vision and image processing to assess the performance of image segmentation methods. The equation of accuracy can be computed as:

(13) $${Accuracy}_{score}=\frac{TP+TN}{TP+TN+FP+FN}$$

The Dice coefficient is a widely used metric for evaluating the performance of image segmentation algorithms, including those used for nuclei segmentation. It measures the similarity between two sets of binary data, such as the ground truth segmentation and the segmentation produced by an algorithm. The Dice coefficient is a valuable metric for nuclei segmentation because it considers the number of nuclei correctly identified and the level of overlap between ground truth and model segmentation results. The equation Dice score can be computed as:

(14) $${Dice}_{score}=\frac{2\left|x\cap y\right|}{\left|x\right|+\left|Y\right|}$$

The Jaccard coefficient, also known as the Jaccard index or Intersection over Union (IoU), is another widely used metric for evaluating image segmentation algorithms, including those used for nuclei segmentation. Intersection size divided by union size is used to determine how similar two sets of binary data are. In nuclei segmentation, the Jaccard coefficient is often used to compare the overlap between the ground truth and the algorithm’s segmentation. A higher Jaccard coefficient indicates better segmentation accuracy, indicating greater overlap between the two datasets. The equation of the Jaccard score can be computed as:

(15) $${Jaccard}_{score}=\frac{\left|x\cap y\right|}{\left|x\right|+\left|Y\right|-\left|x\cap y\right|}$$

Based on the evaluation of the six representative image patches extracted from all of the slide images, in particular, three were selected from the RUMC dataset, including three types of prostate cancer images, and three patch MoNuSeg were selected from the dataset, including multi-organs histopathological images of liver, breast, prostate, and stomach cancer. These six patch images were used for the visual and qualitative evaluation of the results of the partition in various machine learning algorithms.

We evaluated the performance of Random Forest, SVM, K-means, and Logistic Regression methodologies for nuclei segmentation from histopathology images of prostate cancer tissues. To provide ground-truth labels for comparison, a pathologist with experience in the field manually annotated the images. The performance of the segmentation algorithms was evaluated using the Dice coefficient and the Jaccard Index.

The results show that Logistic Regression with VGG-16 feature methodologies achieved high accuracy in segmenting the nuclei, as shown in Table 1. The Logistic Regression algorithm achieved a 74.24 Dice coefficient and 55.61 Jaccard Index. In terms of handcrafted features, SVM achieves the highest accuracy. Still, Logistic Regression achieves higher segmentation performance results where SVM, Dice coefficient 68.90, Jaccard coefficient 52.04, accuracy 98, respectively, and for Logistic Regression, Dice coefficient 68.32, Jaccard coefficient 53.81, and accuracy 96.10, respectively, as shown in Table 2. The Logistic Regression algorithm outperformed the Random Forest algorithm in terms of Dice coefficient and Jaccard coefficient, indicating that Logistic Regression with VGG-16 feature methodology is more accurate and precise for nuclei segmentation.

**Figure 11:**
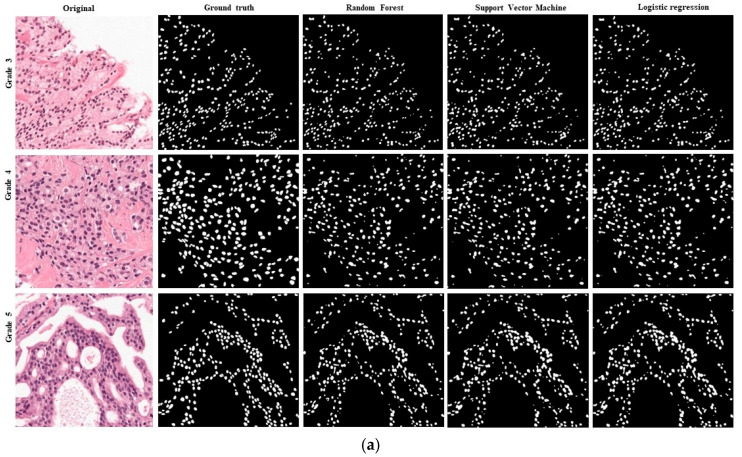

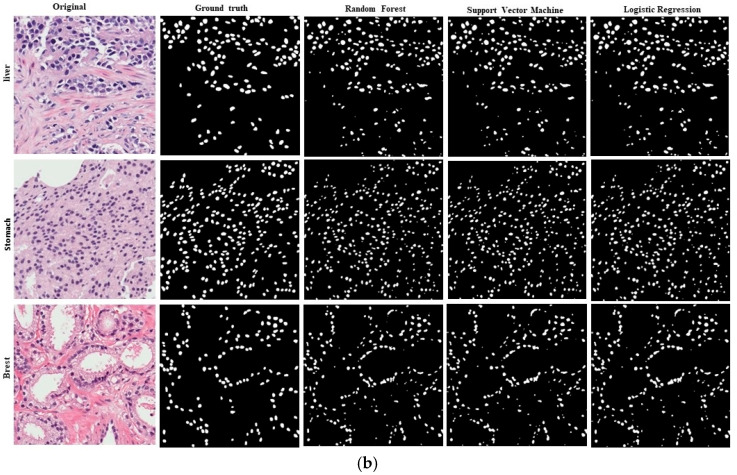
Nuclei segmentation results are based on various artificial machine learning algorithms trained with VGG-16 features. The annotated samples were used for evaluation. The resulting images of each algorithm are shown in their respective row. (**a**) Results of the Radboud University Medical Center. (**b**) Results of the MoNuSeg dataset.

**Figure 12:**
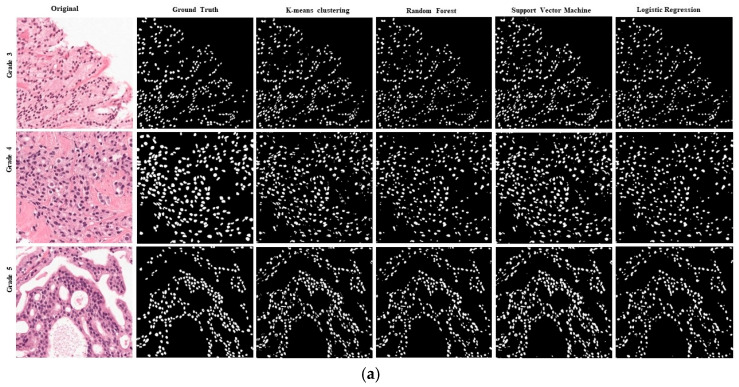

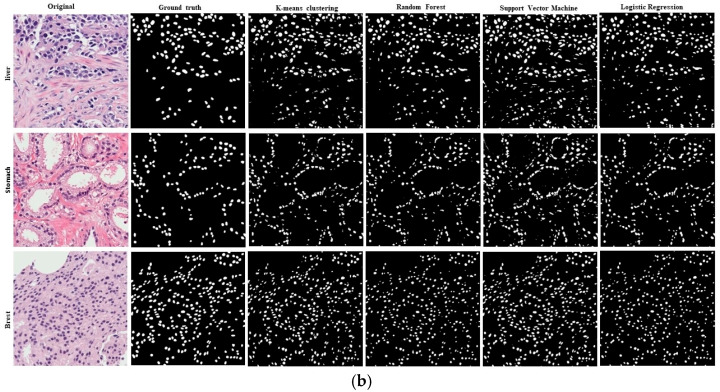
Results of nuclei segmentation based on a variety of artificial machine learning algorithms trained with handcrafted features. The annotated samples were used for evaluation. The resulting images of each Algorithm are shown in their respective row. (**a**) Results of the Radboud University Medical Center. (**b**) Results of the MoNuSeg dataset.

We applied to the Radboud University Medical Center and Yonsei Severance Hospital datasets in this experiment. Figure 11 shows the visual representation of nucleus segmentation based on the CNN feature, and Figure 12 demonstrates the visualization result based on the handcrafted feature. In this experiment, we employed two separate datasets with various ethnic origins. We performed stain normalization to standardize the tissue appearance and ensure our comparison samples were reliable. Very poor sample preparation or digitalization can harm segmentation. In this study, we introduced a small data histopathology image dataset of P.C. with more than 10,000 nuclei annotations. We modified the VGG-16 model for feature extraction. We achieved D.C. and J.C. scores of 74% and 55% for the Logistic Regression model with the VGG-16 feature, respectively. Several researchers have developed segmentation models using publicly accessible cell nuclei datasets from multiple organs and performed comparative analyses. However, this study compares different ML algorithms based on handcrafted and CNN features. Our results demonstrate that Logistic Regression with the CNN feature is an effective method for nuclei segmentation from histopathology images, achieving high accuracy and consistency. Results from these studies significantly improve the accuracy and effectiveness of cancer diagnosis and treatment using automated histopathology image analysis.

## 5. Discussion

This study compared the nucleus segmentation using the handcrafted and CNN-based features extracted with a pre-trained VGG-16 model. Experimental results demonstrated that Logistic Regression combined with CNN features achieved the highest partition performance, with Dice coefficients of 74.24, a Jaccard index of 55.61, and an accuracy of 96.90%. In comparison, traditional machine learning methods such as Random Forest, SVMs, and K-means produced low partition performance on handcrafted and CNN-based feature sets. These results can be attributed to many factors. First, CNN-based features, especially extracted from deep layers of VGG-16, have captured complex textures, spatial, and structural information from histopathology images. Unlike handcrafted methods, which rely on predetermined filters and may not be well-suited to diverse histological patterns, CNN features learn relevant representations during training, making them stronger for complex tissue types such as prostate cancer. Second, Logistic Regression improved other machine learning algorithms due to its strength in binary classification, which works with relatively small training datasets. Since nucleus segmentation is considered a binary classification problem (nucleus vs. background), Logistic Regression was well suited to handle this task efficiently. Compared to more complex models, its simplicity and low computational costs also contributed to its stable and accurate performance, especially when the input feature space is already adapted through CNN-based extraction. In contrast, while the SVM performed properly with handicraft features, it did not cross the logistic region in CNN-based feature settings. This can be due to the sensitivity of SVM’s kernel and parameter tuning, especially when applied to high-dimensional feature vectors from the deep network. Similarly, as an unheard-of clustering algorithm, this partition work lacked supervision to perform competitively in this segmentation task.

Additionally, we implemented stain generalization as a preprocessing step to address the issues of color variation in the dataset (RUMC and MoNuSeg), which helped improve model generalization. Despite working with limited annotated patches during the evaluation (six patch images, three from each dataset), the division’s results were visually and quantitatively promising. This study indicates that high partitions may be accurate by maintaining deep CNN features with logistics such as a simple, effective logistic region, such as a classifier. Conclusion models emphasize convenience representation, while complexity emphasizes the importance of quality, especially in medical imaging functions with limited data. The future work will focus on expanding the dataset size and discovering end-to-end deep learning models or hybrid approaches.

## 6. Conclusions

This study aimed to evaluate machine learning methods for segmenting histopathological nuclei images through CNN features and handcrafted Features. However, the best segmentation results were achieved using the CNN-based features and Logistic Regression algorithm, with values of 74.24 and 55.61 for the Dice coefficient and Jaccard coefficient, respectively, shown in Table 1, and a Dice coefficient of 68.32 and Jaccard coefficient of 53.81 for the handcrafted feature, shown in Table 2. It outperformed all standard segmentation algorithms we tested through the various machine learning algorithms. In the future, we will introduce a large prostate cancer dataset and apply a deep learning method based on AI. Future work will also explore a segmentation technique based on machine learning. Although the method reported here significantly improves existing segmentation models, further exploration is needed for better synthesizing touching nuclei using a multi-channel deep learning network based on U-Net.

Finally, our experimental results demonstrate that the segmentation technique can perform better through CNN features and Logistic Regression algorithms. Using a real-time system, we can devise more efficient treatment plans in computational pathology.

## Figures and Tables

**Table 1:** A comparative analysis experiment of the segmentation result based on VGG-16 features.

Value (Confidence Interval)	Feature Extraction	Average Dice Coefficient	Average Jaccard Index	Accuracy
Random Forest	CNN	69.22 (58.1–80.3)	53.46 (43.6–69.1)	93.7 (90.9–95.1)
SVM	CNN	65.72 (55.8–75.4)	49.38 (38.7–60.5)	97.8 (95.1–98.9)
Logistic Regression	CNN	74.24 (59.6–79.6)	55.61 (43.1–66.1)	96.9 (95.9–97.1)

**Table 2:** A comparative analysis experiment of segmentation results performed based on handcrafted features.

Value (Confidence Interval)	Feature Extraction	Average Dice Coefficient	Average Jaccard Index	Accuracy
Random Forest	Handcraft	67.19 (52.2–78.7)	51.34 (35.4–64.9)	92.7 (92.6–96.4)
SVM	Handcraft	68.90 (44.5–80.0)	52.04 (28.7–66.5)	98.0 (94.1–98.9)
K-Means	Handcraft	61.23 (38.5–81.5)	46.66 (29.9–68.8)	……
Logistic Regression	Handcraft	68.32 (42.9–81.1)	53.81 (28.1–68.1)	96.1 (91.9–96.1)

## Data Availability

Publicly available. We used publicly available data from a public dataset. In The Netherlands, Radboud University Medical Center prepared the public dataset https://www.kaggle.com/code/tachyon777/panda-tachyon-introduction. Another publicly accessible dataset called Multi-organ Nucleus Segmentation (MoNuSeg) was acquired. It may be accessed at https://monuseg.grand-challenge.org/Data.
